# Improving Genomic Prediction in Cassava Field Experiments Using Spatial Analysis

**DOI:** 10.1534/g3.117.300323

**Published:** 2017-11-07

**Authors:** Ani A. Elias, Ismail Rabbi, Peter Kulakow, Jean-Luc Jannink

**Affiliations:** * Department of Plant Breeding and Genetics, Cornell University, Ithaca, New York 14853; † International Institute of Tropical Agriculture, Ibadan, 200001 Nigeria; ‡ United States Department of Agriculture-Agricultural Research Service, Robert W. Holley Center for Agriculture and Health, Ithaca, New York 14853

**Keywords:** cassava, genomic selection, spatial kernel, predictability, GenPred, Shared data resource

## Abstract

Cassava (*Manihot esculenta* Crantz) is an important staple food in sub-Saharan Africa. Breeding experiments were conducted at the International Institute of Tropical Agriculture in cassava to select elite parents. Taking into account the heterogeneity in the field while evaluating these trials can increase the accuracy in estimation of breeding values. We used an exploratory approach using the parametric spatial kernels Power, Spherical, and Gaussian to determine the best kernel for a given scenario. The spatial kernel was fit simultaneously with a genomic kernel in a genomic selection model. Predictability of these models was tested through a 10-fold cross-validation method repeated five times. The best model was chosen as the one with the lowest prediction root mean squared error compared to that of the base model having no spatial kernel. Results from our real and simulated data studies indicated that predictability can be increased by accounting for spatial variation irrespective of the heritability of the trait. In real data scenarios we observed that the accuracy can be increased by a median value of 3.4%. Through simulations, we showed that a 21% increase in accuracy can be achieved. We also found that Range (row) directional spatial kernels, mostly Gaussian, explained the spatial variance in 71% of the scenarios when spatial correlation was significant.

Estimation of breeding value for targeted genotypes is the main aim of a breeding experiment. Error in this estimation due to field heterogeneity is a critical factor while conducting evaluations in the field. Efficient experimental designs have been developed to mitigate this issue. For example, blocking can be used to group experimental units (plots) in less heterogeneous space based on prior information about the field. Efficiency of blocking primarily depends on the assumption that plot variation within a block is small compared to that among blocks (Gusmao 1986). However, this might not be true, either due to the large size of blocks, or bad alignment of the blocks with the field variability (Stroup *et al.* 1994). Spatial variation within a block can mask the genotypic effect, and result in reduced accuracy in estimation and wrong ranking of genotypes.

The primary idea of accounting for spatial dependency was proposed by Papadakis (1937) using the nearest neighbor (NN) approach. The NN approach was a simple method where spatial dependency was assumed to be discontinuous beyond the neighbors in perpendicular directions. Wilkinson *et al.* (1983) modified this model using an iterative approach. Schwarzbach (1984) developed another variation of iterative NN in which spatial adjustments were made only in one direction, unlike that in the model proposed by Papadakis (1937).

The random field (RF) approach in spatial experiments provides more accurate estimates of treatment contrasts than NN approaches. The spatial heterogeneity can be modeled as a random process. When spatial coordinates index the random variables in this process it is called a RF (Chung 2007; Adler 1981). The RF can be isotropic, meaning the spatial heterogeneity lacks any preferred direction, or it can be directional, assuming the heterogeneity follows Range or Column directions. The spatially related variables in RF can be modeled using a correlation structure. The realization of these spatial variables (*e.g.*, temperature and soil moisture) in the RF (Cameletti *et al.* 2013) can be estimated as a function of distance. These realizations can be used to make inferences about the process, and also for spatial prediction (Zimmerman and Harville 1991). The RF approach can be used to analyze data with heterogeneous plot size and shape (Matérn 1986), in agricultural fields having spatial variation along all dimensions, (Schabenberger and Pierce 2010; Gonçalves *et al.* 2007; Stroup *et al.* 1994), and in conjunction with blocking effects.

Use of RF in agricultural field experiments was initiated by Gleeson and Cullis (1987), who proposed to sequentially fit a low-order autoregressive-integrated-moving-average (ARIMA) correlation structure to the plot errors in one direction. This model increased trial efficiency. The single directional spatial trend analysis was analogous to time series analysis where spatial points corresponded to time points. Later, Cullis and Gleeson (1991) modified this model to use both Range (Row) and Column directions in a regularly spaced field. For quantitative traits in agriculture, first order separable (separate functions for Row and Column) autoregressive (AR) structure of residuals as direct product (AR1 × AR1) was considered as an appropriate initial model for spatial analysis (Gilmour *et al.* 1997). AR structure is a special case of the more general ARIMA structure.

A generalization of AR is the Power correlation structure. AR assumes that the points are equidistant, whereas the Power structure accounts for nonequal distance, and continuity of values under study (Piepho *et al.* 2015). In the Power structure, the correlation is raised to power of the actual distance between points unlike that in AR where powers of 1, 2, 3, etc. are used. AR and related structures are commonly used in agricultural field evaluations. However, other forms of dependency should also be tested.

In modern plant breeding, a large number of test genotypes are evaluated, and they can often not be replicated within an experimental field due to limitation in resources. Therefore, only check genotypes are replicated and randomly assigned to the field in blocks, or Ranges and Columns. Genomic selection (GS) models can be used to mitigate the lack of replication while evaluating these genotypes: GS models use a genomic relationship matrix based on genetic markers, so that phenotypic information is shared across related genotypes (Goddard and Hayes 2007). Using all marker data as predictors of performance can consequently deliver more accurate estimations (Jannink *et al.* 2010). In this paper, we discuss the use of GS models with a RF component to account for spatial variation in order to reduce the error in estimating breeding value.

Gilmour *et al.* (1997) distinguished natural, extraneous, and global sources of experimental variation. Natural variation comes from soil and/or topographic features. This variation can be taken into account by the GS model using a spatial correlation structure. Extraneous variation is due to experimental operations (*e.g.*, direction of field tillage), and can be modeled using random effects of Range and Column in the model. Global variation is modeled as caused by variation in genotypes. Selection of genotypes after accounting for the potential spatial dependency can provide better estimates of the genotypic effect and modify ranking of the genotypes (Duarte and Vencovsky 2005).

In this study, we illustrated the application of three parametric correlation structures, Power, Gaussian, and Spherical for spatial analysis. We used a cross-validation (CV) method to determine the spatial dependency instead of relying on a semivariogram. CV was used because improvement in predictability was our primary purpose. Simulation studies were also conducted to explore the behavior of models given different forms and importance of spatial variation. Finally, we provided R-based functions to automate real data analysis and simulations.

We used cassava (*Manihot esculenta* Crantz), which is a staple food in much of tropical Africa as the study organism. Cassava is an important crop for food security in Africa due to its drought tolerance, ability to grow in marginal soil, and flexible harvest period (Fresco 1986; El-Sharkawy 2006). This is the main source of calories for half a billion people in Africa (FAO 2004).

## Materials and Methods

### Materials and design of experiment

The International Institute of Tropical Agriculture (IITA) conducted experimental field trials in 2013 and 2014 using cassava breeding populations in Ibadan, Ikenne, and Mokwa in Nigeria. The clones for the trials consisted of the IITA Genetic Gain (GG) population comprised of historically important, mostly advanced genotypes including those derived from the West African gene pool, the Tanzania interspecific hybridization program, and hybrids from the Latin American gene pool (Wolfe *et al.* 2016; Ly *et al.* 2013). We also used data from cycle 1 (C1; progeny of GG), and cycle 2 (C2; progeny primarily of C1), and the GG clones themselves in a preliminary yield trial (PYT). In summary, 83 parents from GG population gave rise to 2187 progenies for C1. Later, 84 C1 and 13 GG clones (total 97) were selected as parents giving rise to 2466 progenies for C2.

The fields were partitioned into Ranges running from North to South and Columns running from East to West. The genotypes were assigned to a field in a randomized design using replicated check genotypes (1–10 check genotypes). Unreplicated test genotypes belonging to the same family were assigned to adjacent plots in a Range in C1 and C2 trials. Check genotypes were used in C1 and C2 trials, and they were assigned to a Range in such a way that every Range had two checks, except when the field layout required particularly small Ranges. Exceptions had no checks or up to four checks in a Range. For the PYT, checks were not used and test genotypes were replicated twice in a randomized block design. Details on the dimensions of field and plot can be found in Table 1, and the information on number of genotypes and replications can be obtained from Table 2. Plots were arranged in a serpentine fashion starting from the first Range. Plots were rectangular in shape, with their longer edge shared across the Ranges. This arrangement means that the distance between two adjacent Ranges was shorter than that between two adjacent Columns.

**Table 1:** Details of trials used in real data analysis

Year	Cycle	Plot Dimension	Location	Field Dimension	#Plots
2013	C1	5 × 1	Ibadan	24 × 33	736
			Ikenne	16 × 54	855
			Mokwa	8 × 116	858
2014	C1	5 × 4	Ibadan	19 × 18	293
			Ikenne	19 × 18	330
			Mokwa	19 × 18	329
	C2	5 × 1	Ikenne	10 × 46	444
			Mokwa	20 × 23	432
	PYT	10 × 1	Ibadan	8 × 26	176

Plot dimension is expressed as length × width, where length is the number of plants in a row, and width is the number of rows in a plot. Field dimension is expressed as the number of Ranges × number of Columns in a field. Finally, #Plots gives the number of plots planted.

**Table 2:** Comparison between linear models Base and Model 1 for DM, FYLD, SHTWT, and HI

Data	Trait	Model	Variance			h2	Spatial Structure	LLk	*χ*-Square	Increase in pCOR (%)	Decrease in pRMSE (%)
			$\sigma_{g}^{2}$	$\sigma_{s}^{2}$	$\sigma_{r}^{2}$						
Ibadan_2013_C1	DM	Base	16.155	NA	6.127	0.28	NA	−949.14	3.82	2.1	0.8
	(511 & 485)	Model1	15.604	0.802	5.913	0.29	Gaus - Range	−947.23	(0.05)		
							(*ϕ* = 1)				
	SHTWT	Base	10.185	NA	11.888	0.58	NA	−1284.39	17.474	5.4	1.4
	(660 & 631)	Model1	9.022	1.729	11.875	0.59	Gaus - Range	−1275.65	(2.90E−05)		
							(*ϕ* = 8)				
Ibadan_2014_PYT	DM	Base	15.866	NA	15.418	0.39	NA	−310.06	39.724	27.8	9
	(148 & 80)	Model1	18.692	6.404	8.512	0.28	Gaus - Range	−290.2	(2.9e−10)		
							(*ϕ* = 2)				
	SHTWT	Base	23.556	NA	101.2	0.73	NA	−433.64	10.782	23.1	4
	(151 & 81)	Model1	27.546	16.702	84.024	0.68	Sph - Range	−428.25	(0.001)		
							(*ϕ* = 0.5)				
Ibadan_2014_C1	FYLD	Base	879.063	NA	49.264	0.46	NA	−1026.56	4.084	1.1	0.1
	(286 & 266)	Model1	883.751	40.685	38.905	0.46	Gaus - Isotropic	−1024.52	(0.043)		
							(*ϕ* = 73)				
	HI	Base	0.018	NA	0	0.4	NA	518.78	3.894	2.4	1
	(282 & 265)	Model1	0.017	0.031	0	0.37	Gaus - Range	520.73	(0.048)		
							(*ϕ* = 140)				
Mokwa_2013_C1	DM	Base	15.983	NA	8.862	0.38	NA	−1106.55	8.964	1.6	0.7
	(571 & 537)	Model1	15.536	1.455	8.602	0.37	Power - Range	−1102.07	(0.003)		
							(*θ* = 0.7)				
	FYLD	Base	2.469	NA	18.865	0.84	NA	−1477.95	4.286	4.4	0.4
	(734 & 694)	Model1	1.649	1.113	19.218	0.86	Gaus - Range	−1475.81	(0.038)		
							(*ϕ* = 4.5)				
Mokwa_2014_C2	DM	Base	13.946	NA	9.891	0.35	NA	−513.17	7.294	5.2	1.3
	(260 & 239)	Model1	13.171	1.13	9.215	0.37	Gaus - Range	−509.52	(0.007)		
							(*ϕ* = 1.5)				
	HI	Base	0.008	NA	0.012	0.63	NA	473.95	6.736	6.2	1.5
	(310 & 286)	Model1	0.008	0.004	0.011	0.61	Gaus - Range	477.32	(0.009)		
							(*ϕ* = 18.5)				
	SHTWT	Base	1.743	NA	37.669	0.91	NA	−754.11	4.418	8	0.6
	(324 & 300)	Model1	1.943	159.034	36.727	0.9	Gaus - Range	−751.9	(0.035)		
							(*ϕ* = 111)				
Ikenne_2013_C1	DM	Base	25.097	NA	4.387	0.31	NA	−1205.86	7.804	1.3	0.3
	(627 & 611)	Model1	26.914	0.903	2.919	0.32	Gaus - Isotropic	−1201.96	(0.005)		
							(*ϕ* = 9)				
	HI	Base	0.007	NA	0.012	0.51	NA	1167.01	3.04	1.2	0.7
	(757 & 736)	Model1	0.007	0	0.012	0.51	Gaus - Isotropic	1168.53	(0.081)		
							(*ϕ* = 55)				
	SHTWT	Base	13.31	NA	17.192	0.65	NA	−1650.46	3.932	1.4	0.2
	(781 & 753)	Model1	13.581	0.672	16.467	0.64	Power - Column	−1648.49	(0.047)		
							(*θ* = 0.9)				

Under the Trait, the number of observations and unique genotypes analyzed is given in brackets. Variance of zero indicates that variance was <1e−03. Narrow sense heritability (h2) is calculated from the BLUP values of genotypes and genotypic variance. Spatial structure is given with the direction and the parameter value in brackets. Chi-square statistic is calculated from the log likelihood values (LLk) of the Base and selected Model 1 is given with p-value in brackets. The table shows results from trial-trait analysis with significant improvement in fit of Model 1 over Base at *α* = 0.1. Percentage change in pCOR and pRMSE for Model 1 compared to Base after CV are given.

For the current study, four agronomic traits were evaluated: fresh weight of storage roots (FYLD) and root dry matter content (DM), fresh weight of shoots (SHTWT), and harvest index (HI). The DM is the percentage of the root that is not water. The FYLD is the fresh root weight measured in kilograms. The SHTWT is the total fresh weight of harvested foliage and stems measured in kilograms. The HI is the proportion FYLD to the total harvested weight (FYLD + SHTWT) (Ly *et al.* 2013). Genotyping of single nucleotide polymorphic (SNP) markers was done as described by Wolfe *et.al*. (2016).

The relatedness of genotypes (including check and test) within a field study was calculated based on the additive relationship matrix using all the markers with >1% minor allele frequency. The relationship matrix was calculated as described by Endelman and Jannink (2012) using the A.mat function from the package “rrBLUP” in R. The function uses the Method 1 of VanRaden (2008).

### Statistical models

**Figure 1:**
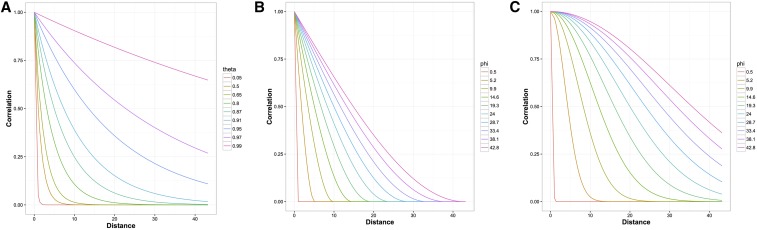
Spatial correlation with distance (meters) using different structures and standardizing parameters—an illustration. (A) Power; (B) Spherical; (C) Gaussian.

To explore the spatial correlation, we used three correlation structures: the generalized autoregressive or Power, Gaussian (Gaus), and Spherical (Sph). The correlation structures were calculated as follows:

$$Power=\theta^{D_{n\times n}}$$,

$$Gaus=exp\left(\frac{-D_{n\times n}^{2}}{\phi^{2}}\right)$$,

$$Sph=\left[1-1.5\left(\frac{D_{n\times n}}{\phi }\right)+0.5\left(\frac{D_{n\times n}^{3}}{\phi^{3}}\right)\right]if\;\left(D_{ij}\leq \phi \right);else\;0$$,

where *θ* is the standardizing parameter for Power whose values range from 0 to 1; *ϕ* is the standardizing parameter for other RF structures whose values can range from 0 to the maximum distance between any two plots in the field; *D* is the Euclidean distance matrix and *n* is the number of observations in the dataset; and *i* and *j* are indices to plot coordinates. Depending on the value of the standardizing parameter, the correlation expressed by these structures decays more or less rapidly with distance (Figure 1). The larger the standardizing parameter, the slower is the decay. The correlation value for Power and Gaus structures reaches zero asymptotically. For the spherical, the correlation is conditionally limited to zero based on the value of standardizing parameter.

The distance matrix was calculated taking into consideration the plot dimension. Therefore, the distance between plots in any two Ranges (range distance) and Columns (column distance) was calculated as the number of Ranges multiplied by the width of the plot, and the number of Columns multiplied by the length of the plot. The distance between plots that were diagonal to each other was calculated as the hypotenuse of a triangle based on the Pythagorean theorem:

$$D=\sqrt{range\;distance^{2}+column\;distance^{2}}$$.

We considered three scenarios of potential spatial correlation. First, the correlation was assumed to be isotropic, and the distance was calculated in Range, Column, and diagonal directions. In the second and third scenarios, correlation was assumed to be Range or Column directional as special cases of anisotropy. Here, either Range or Column distance was taken into consideration while calculating the distance matrix. The model used was:

$$Y_{n\times 1}=\mu +Z_{n\times g}g_{g\times 1}+\epsilon$$, (1)

$$\begin{array}{ccc} & \epsilon =s_{n\times 1}+r_{n\times 1} & \\ s∼N\left(0,S\sigma_{s}^{2}\right) & & r∼N\left(0,I\sigma_{s}^{2}\right)\\ & g∼N\left(0,K\sigma_{g}^{2}\right), & \end{array}$$

where **Y** is the response variable (*e.g.*, DM); ***μ*** is the general mean; **Z** is the design matrix for genotypic effect, *n* is the number of observations, and *g* is the number of unique genotypes in the data; ***g*** is the vector of genotypic effect; ***s*** and ***r*** are the vectors of spatial effect and residual error; **K** is the genomic relationship matrix (here, the additive relationship matrix); and **S** is the spatial correlation matrix; **I** is the identity matrix.

A ten-fold CV repeated five times was used to calculate the predictability. Genotypes were separated into folds at random, ensuring that the training and test datasets did not contain the same genotypes. Because the genotypes were random, the specific plots included in each fold were also random. Model 1, using various spatial structures, was compared to a base model (here after called Base) having no spatial component. The best model was chosen as the one having the lowest prediction root mean squared error (pRMSE) between observed (**Y**) and calculated (**Ŷ**) response values for the test dataset. The calculated response (**Ŷ**) was

$${\hat{Y}}_{n\times 1}=\mu +Z_{n\times g}{\hat{g}}_{g\times 1}+I_{n\times n}{\hat{s}}_{n\times 1},$$

where *n* is the number of observations in the test data; the design matrix for genotypic effect in test data are **Z** with the dimension *n × g*, where *g* is the number of unique genotypes in the full dataset (training + test); $\hat{g}$ is the best linear unbiased prediction (BLUP) of genotypes calculated from the model on fitting the training data; the design matrix for spatial effect in test data are **I** with dimension *n × n*; and $\hat{s}$ is the BLUP of the spatial effect. To calculate the response variable for the base model, everything else was the same but the spatial component was removed. Relative reduction in pRMSE for Model 1 was calculated as the ratio of difference in pRMSE to Base pRMSE. The prediction correlation (pCOR or accuracy) was also recorded for the best model as the correlation between observed and calculated response values for the test dataset. Relative increase in pCOR was calculated as the ratio

$$\frac{pCOR_{Model1}-pCOR_{Base}}{1-pCOR_{Base}}$$.

The spatial components accounted for by Model 1 assume smooth decay of correlation with distance. Field operations, however, can lead to discontinuities between Ranges or Columns not well fitted by Model 1 [the extraneous error of Gilmour *et al.* (1997)]. To test and account for such error structures, we added a random effect of Range, Column, or both. These effects were added to the Base model if it was the best model after CV. The model with Range and Column effects is called Model 2. A full Model 2 with both Range and Column effect is

$$Y_{n\times 1}=\mu +Z_{n\times g}g_{g\times 1}+Z2_{n\times ra}ra_{ra\times 1}+Z3_{n\times cl}cl_{cl\times 1}+\epsilon$$, (2)

$$\mathrm{ra}∼N\left(0,I\sigma_{ra}^{2}\right)\;\mathrm{cl}∼N\left(0,I\sigma_{cl}^{2}\right)$$,

where **Z2** with the dimension *n ×* *ra* is the design matrix for the range effect, ***ra***; **Z3** with dimension *n ×* *cl* is the design matrix for the column effect, ***cl***.

The data were fitted using the “regress” package in R v. 3.2.5 using restricted log likelihood. A Chi-square test was used to test the significance of the additional variance component in the selected model compared to the base. Since the $\chi^{2}$ follows a mixture distribution, the significance threshold with one degree of freedom was taken as 2.706 at *α* value of 0.1 (Stram and Lee 1994).

$$\chi^{2}=2\left(\text{llk}\;\text{of}\;\text{model}-\text{llk}\;\text{of}\;\text{base}\right)$$

$$\chi^{2}∼\frac{1}{2}\chi^{2}\left(0\right):\frac{1}{2}\chi^{2}\left(1\right),$$

where *llk* is the log likelihood, and “model” is the selected model adding either the spatial error component and/or the extraneous error component to the base model. The threshold value was changed with degrees of freedom. For example, on comparing Model 1 with a full Model 2 having two additional components, the threshold was taken as 4.605 at *α* value of 0.1. Heritability was calculated based on the approach introduced by Cullis *et al.* (2006) as follows:

$$h^{2}=1-\frac{{\hat{V}}_{BLUP\;difference}}{2{\hat{\sigma }}_{g}^{2}},$$

where ${\hat{V}}_{BLUP\;difference}$ is the variance of difference between pairs of genotypic BLUPs; ${\hat{\sigma }}_{g}^{2}$ is the estimated variance of genotypes. The datasets and SNP file used for performing this study can be found in the following link:datasets.

### Simulation studies

We conducted a simulation study to (i) test the validity of the GS-spatial model in reducing the error and in correctly partitioning genotypic variance, and (ii) evaluate the performance of this model as a function of the different parameters involved. A dataset containing 829 genotypes, including 11 check genotypes and their field coordinate information, was used to start the simulations. Genotypic effects were simulated with zero mean and unit variance and without using the relationship matrix.

We simulated ratios of spatial to total error variance ranging from 0.3 to 0.9, and of genotypic to total phenotypic variance also ranging from 0.3 to 0.9. The combination of genotypic and spatial error ratio values was used to determine the variance of spatial and residual error effects. Genotypic ratio determined the variance in total error, which was then partitioned into spatial and residual based on their ratio. The variance indicated the spatial heterogeneity while different standardizing parameters (*θ* or *ϕ*) defined the coverage of correlation.

Two different correlation structures were used to simulate the spatial effect: Power and Gaussian. These two were chosen as they were the most dissimilar among structures used in this study. For Power, *θ* values ranged from 0.2 to 0.8 and for Gaus, *ϕ* values ranged from 0.5 to 60.5. We believe these ranges cover plausible values that might be encountered in practice (Figure 1). The correlation was calculated on the assumption that plot dimension was 2 × 1.

Three scenarios of genotype replication were considered. First, a dataset with minimum replication contained only replicated checks with all the test genotypes represented once. Second, 50% of the test genotypes were replicated twice in addition to the presence of replicated checks. In a third scenario all test genotypes were replicated twice.

Data were analyzed with all three spatial models and the base model. The best model was selected as the one with the lowest RMSE, where error is the deviation between the true simulated genotypic effect and the estimated genotypic effect, and highest accuracy (correlation) between estimated and realized genotypic effects.

### Automation of real data analysis, simulation of data, and its analysis

Functions were written to automate the real data analysis and simulations using algorithms described above. For the real data analysis, the minimum requirements for the function are a .csv file having field coordinates (Ranges and Columns), trait(s), and genotypes, and a genotypic relationship matrix based on marker or pedigree data. Providing a plot dimension (width × length to calculate distance between Ranges and Columns) can help to increase the accuracy of the model. The function can take care of the initial processing of the data, including removal of missing values for a particular trait, matching the genotypes with those in the relationship matrix, and removal of potential outlier points that have a residual 2.5 times the residual SD (after testing using the base model). Outlier data points are removed because they can affect the spatial dependency in the field. The output of the function will be saved in the working directory of R. The output will contain the predictions for genotype and spatial effect, pRMSE and pCOR values. Given that the function fits many models, some models fail, and these are trapped by the “try” function in R. The number of such failed models is returned. Finally, summaries of the base and the selected model, including the standardizing parameter value (if the selected model is different from the Base), is returned. For the simulations, a dataset with genotypes and field coordinates is to be given, along with a vector of standardizing parameters, fraction value for spatial to total error variance, genotypic ratio or heritability value, and plot dimension. The output contains .csv files of RMSE and COR between simulated and predicted genotypic, spatial, and residual effects, fraction of spatial error, heritability, and number of models failed. The functions are available in the following folder: ftp://ftp.cassavabase.org/manuscripts/Elias_et_al_2017_spatial.zip.

### Data availability

**Figure 2:**
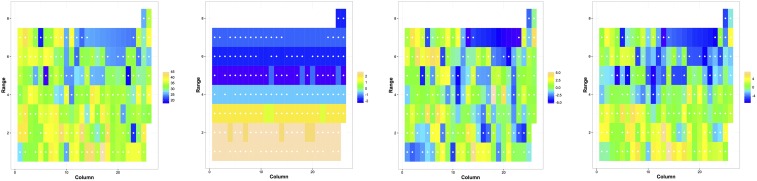
Original observation (column 1), spatial BLUP (column 2), residual from Model 1 (column 3), and residual from Base (column 4) for dry root weight (DM) for Ibadan_2014_PYT. Plots are rectangular, and white dots inside each plot indicate the plots whose observation was used. Missing values were interpolated linearly for visualization. These plots do not represent the actual dimension of those in the field, but used as placeholders in order to visualize the trends in effects.

Supplemental Material, Tables S.1–S.6 in File S1 contains ANOVA tables on analyzing the simulated data. Figure S.1 in File S1 indicates the variation in spatial to total error variance for simulated data. Figure S.2 in File S1 shows the original observation, spatial BLUP, residual from Model 1 and Base for all the trial-traits mentioned in Table 2 except for that in Figure 2.

## Results

### Real data studies

For 39% of the trial–trait combinations, Model 1 fit significantly better than Base at an *α* value of 0.1 (Table 2). All but one of these scenarios showed reduction in error variance, up to 44%, compared to residual error variance from Base. One instance (Mokwa_2013_C1) FYLD showed a marginal increase in error variance. This instance also showed a reduction in genotypic variance of 33%. The spatial kernel explained from 2 to 80% of the total phenotypic variance when spatial dependency was significant. Total phenotypic variance remained unchanged on comparing the values between Base and Model 1 in 50% of instances. In 36% of the scenarios, a marginal increase of phenotypic variance, with a median relative value of 3.6% was observed. This increase was due to a marginal increase in genotypic variance, and contribution of spatial variance. For the remaining two cases—HI in Ibadan_2014_C1 and SHTWT in Mokwa_2014_C2—total phenotypic variance increased by 167 and 402% respectively. In Ibadan_2014_C1, the spatial variance contributed to 64% of the total variance, and a 5.5% decrease in genotypic variance was observed. In Mokwa_2014_C2, the genotypic variance increased by 11%, while the spatial variance contributed to 80% of total phenotypic variance in Model 1. A large contribution of spatial variance resulted in increased phenotypic variance in both cases. The increase in phenotypic variance due to the spatial component could be an artifact of the spatial correlation matrix with high standardization parameter.

Heritability of the traits ranged from 0.28 to 0.9 (Table 2). Slight or no modification in heritability was observed when spatial variation was accounted for in the model. An exception was the 28% decrease in heritability observed in the PYT trial for DM.

Predictability as measured by reduction in error between observed and predicted values (pRMSE) was improved by <2% in most cases (Table 2). For Ibadan_2014_PYT DM and SHTWT, pRMSE decreased 9 and 4% respectively. These two cases also exhibited a relative increase in accuracy of 28% and 23%, respectively, while an increase of <10% was observed in the remaining cases.

Adding random Range and Column significantly improved 13% of the models previously fitted by just the spatial kernel, while 28% of datasets where the Base was best were improved by Range and Column effects (Table 3). In general, adding extraneous effects did not help in explaining the phenotypic variance in a model once a significant spatial dependency effect was identified. Nevertheless, we believe that extraneous effects can be expected in an uneven field, and proper use of blocking and orientation of Ranges and Columns should be performed to account for this in the initial designing stage.

**Table 3:** Output on adding extraneous error component to the selected Model 1 or Base for DM, FYLD, SHTWT, and HI

Data	Trait	Model	Variance					h2	LLk	*χ*-square
			$\sigma_{g}^{2}$	$\sigma_{s}^{2}$	$\sigma_{r}^{2}$	$\sigma_{R}^{2}$	$\sigma_{C}^{2}$			
Ibadan_2013_C1	FYLD	Base	7.425	NA	19.791	NA	NA	0.7	−1359.31	21.34
		Model2	6.876	NA	18.171	NA	2.049	0.68	−1348.64	(3.80E−06)
	SHTWT	Model1	9.022	1.729	11.875	NA	NA	0.59	−1275.65	6.48
		Model2	8.686	1.763	11.419	NA	0.645	0.6	−1272.41	(0.01)
	HI	Base	0.005	NA	0.009	NA	NA	0.44	1092.16	7.89
		Model2	0.005	NA	0.009	NA	0.001	0.44	1096.1	(0.005)
Mokwa_2013_C1	FYLD	Model1	1.649	1.113	19.218	NA	NA	0.86	−1475.81	2.88
		Model2	1.362	1.101	18.609	NA	0.824	0.87	−1474.37	(0.089)
	HI	Base	0.005	NA	0.009	NA	NA	0.62	1270.87	10.15
		Model2	0.005	NA	0.009	0.001	NA	0.66	1275.94	(0.001)
Mokwa_14_C1	HI	Base	0.007	NA	0.006	NA	NA	0.59	561.33	3.66
		Model2	0.006	NA	0.005	0	NA	0.54	563.15	(0.055)
Ikenne_2014_C1	DM	Base	31.949	NA	0.719	NA	NA	0.44	−579.46	21.87
		Model2	36.116	NA	0.033	NA	1.379	0.46	−568.52	(2.9e−06)
	SHTWT	Base	721.089	NA	29.191	NA	NA	0.5	−1109.42	3.27
		Model2	752.485	NA	15.792	10.999	NA	0.49	−1107.79	(0.071)

Base is the model having only the genetic variance component. Model 1 has a spatial variance component in addition, and Model 2 has extraneous error component added to the best model selected between Model 1 and Base for a particular trial and trait. Narrow sense heritability (h2) is calculated from BLUP values and genotypic variance. Chi-square statistic is calculated from the log likelihood values (LLk) of the Base/Model 1, and selected Model 2 is given with p-value in brackets. The table shows results from trial-trait analysis with significant improvement in model fit of Model 2 over the best of Base/Model 1 at *α* = 0.1.

The genotypic variance was the largest component of the phenotypic variance in 86% of scenarios. In these trials the selected spatial kernel explained <20% of the variance. There were two cases where the spatial variance was ≥40% of the total spatial + residual variance. Traits DM from Ibadan_2014_PYT and FYLD from Ibadan_2014_C1 indicated that these below ground traits were influenced by the underlying spatial pattern. A striking change in residual pattern can be observed between the residuals from GS-spatial (Figure 2, column 3) and Base (Figure 2, column 4) models for Ibadan_2014_PYT DM. The residuals from the spatial model were distributed randomly, fulfilling the random assumption, whereas those from the Base model followed a spatial pattern. The spatial kernel explained 19% of the total phenotypic variance for DM in Ibadan_2014_PYT with an increase in genotypic variance and a 45% relative reduction in error variance. For Ibadan_2014_C1 FYLD, spatial variance explained only 4% of the total phenotypic variance, and most of the variance was explained by genotype.

The variance of either spatial or residual error converged to a boundary solution in two scenarios—HI in Ibadan_2014_C1 and Ikenne_2013_C1—when significant spatial variance was identified (Table 2). This boundary effect could be an artifact of the model where the error variance is partitioned into two: spatial and residual. Additionally, we found six and two scenarios, respectively, for spatial and residual variance where the values were bound to zero in cases where spatial variance was not found to be significant (result not provided). In such scenarios, lack of estimated spatial variance could also be because the true spatial variance is close to boundary. This phenomenon can be better explained using the simulated data. The estimated spatial variance converged to a boundary solution when the data were simulated with values close to boundary (Figure S.1 in File S1).

The standardization parameter as well as the type of spatial kernel can influence the correlation between estimated spatial and residual effects. The dimension of incidence matrices to explain spatial correlation and residual error is the same. The correlation structure explaining the spatial dependency separates the spatial effect from the residual. In an isotropic function, varying values for effect are expected for each plot, unlike that in a directional function. In a directional function, the same value for the spatial effect is estimated for plots identified as belonging to the same Range or Column. The spatial correlation values of an isotropic function beyond the first pair of plots will be close to zero if the standardization value of the function is small. This scenario can result in a spatial correlation structure similar to the identity matrix used for calculating the residual error. This similarity could contribute to correlation between estimated spatial and residual effects that are otherwise assumed to be uncorrelated.

We used three different spatial kernels to explain spatial dependency. Out of these, the Power function, which is a generalization of the commonly used AR in agricultural experiments, best explained spatial dependency in only 14% of the scenarios (Table 2). The rest of the scenarios were most often (79% of the time) best explained by the Gaussian kernel. Additionally, directional spatial correlation was exhibited in nearly three-fourths of the scenarios, predominantly across Ranges. The center-to-center distance between adjacent plots across two Ranges was lower than that across two Columns, possibly explaining why the Range directional spatial kernel identified dependency even when *ϕ* values were small. Underlying soil characteristics that influenced the performance of cassava could also be a reason for the selection of directional kernels (including Column directional). A clear gradient in spatial dependency in the North-South (N–S) direction was evident in 53% of the scenarios (Figure 2 and Figure S.2 in File S1, column 2). For the remaining scenarios, an uneven gradient can be observed either in N–S or in East–West (E–W) direction. The gradient from spatial variation was similar (Figure 2, Figure S.2 in File S1, column 2, and Table 2) for different traits from the same field. This similarity indicated that the cause of spatial variation was consistent across traits; for example, possibly due to changes in soil properties. However, the influence of soil properties on different traits differed as indicated by the range of variance components (Table 2) and visuals (Figure 2 and Figure S.2 in File S1, column 2).

### Simulation studies

**Figure 3:**
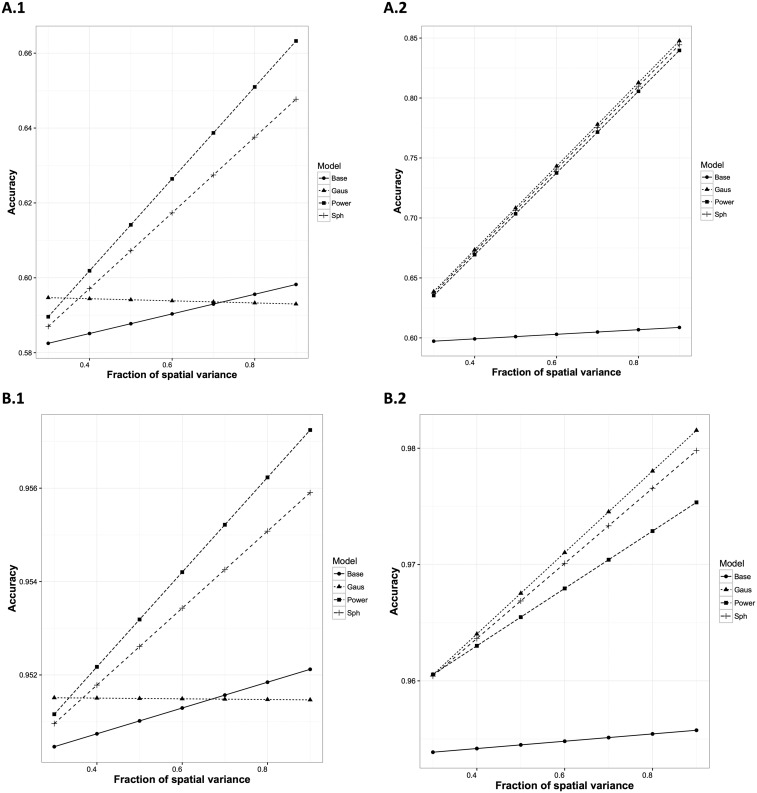
Results from simulation studies using data simulated with Power (column 1) and Gaussian (column 2) spatial kernels. A.1 and A.2 represent data simulated using low genotypic ratio (0.3), while those in B.1 and B.2 represent data simulated with high genotypic ratio (0.9). Accuracy, the correlation of true to estimated genotypic value, is given on the *y*-axis. Note differences in *y*-axis scales across all plots. The *x*-axis represents the fraction of spatial to total error variance (fraSp). The interaction of Model with fraSp is shown by nonparallel lines. ANOVA results are given in Tables S.1–S.6 in File S1.

Results from simulation studies throw light on the importance of identifying a spatial relation in a field with the correct function in order to increase the accuracy in prediction irrespective of the genotypic ratio of the trait (Figure 3 and Tables S.1–S.6 in File S1). It was observed that the Gaussian kernel exhibited the lowest accuracy when the spatial dependency was simulated with the Power function, especially with an increase in the fraction of spatial to total error variance (Figure 3, A.1 and B.1). On the other hand, accuracy when using the Power function on Gaussian simulated data was comparable. However, the rate of increase in accuracy with increase in the fraction of spatial variance was relatively low, with the pattern more noticeable with high genotypic ratio (Figure 3, A.2 and B.2). A similar pattern in accuracy was observed with increase in spatial coverage in both Power and Gaussian simulated datasets (figure not shown). The spatial error could be a confounder for genotypic effect. However, the spatial error can be detected statistically using the correct spatial kernel, and can be removed from genotypic effect. Thus, increased fraction of spatial variation leads to higher accuracy. Furthermore, as the spatial coverage increases, the spatial error becomes more distinct from the residual error, and, therefore, can be better estimated, leading to higher accuracy. Simulation results also showed that the Spherical structure was a robust kernel, irrespective of the underlying spatial pattern, as its accuracy was comparable to the correct kernel in all instances.

Low accuracy values for the Gaussian model, when used to analyze Power spatial pattern, could be because of its dissimilarity with Power or other kernels in explaining spatial correlation (Figure 1). In general, correlation based on all other kernels initially decays rapidly with increase in distance, irrespective of the value of standardizing parameter. In contrast, the Gaussian model, decay is initially slow followed by a rapid decay. The initial slow decay forces plots close to each other to have very similar residuals.

## Discussion

Through the analysis of real and simulated datasets, we showed that predictability of GS models can be improved by adding a non-i.i.d kernel accounting for spatial dependency in trial fields, irrespective of the heritability of the trait. In real data analyses, spatial models increased the prediction accuracy by a median value of 3.4% compared to models lacking a spatial factor. In simulations, scenarios with parameters comparable to those found in real data showed prediction accuracy improvements of up to 21% (Figure 3B.2). Moreover, these improvements were achieved by simultaneously fitting a genotypic and spatial kernel in the same model in a single step. Adjusting for spatial variation is expected to provide better estimates of genotypic BLUP values, thereby facilitating the selection of genotypes.

Use of an AR spatial kernel is very common in agricultural spatial evaluation. A study in rye indicated that use of AR kernels (one- and two-dimensional) did not improve genomic predictability (Bernal-Vasquez *et al.* 2014). We not only considered a generalized AR, Power, but also other forms of spatial kernels, and used an exploratory approach. We used varying values for standardizing parameters while performing this analysis. In addition, we considered isotropic and anisotropic patterns of correlation. All these helped in fitting the complexity and extent of spatial correlation in our fields. A spatial model that is adequate for one scenario may not be suitable for another. This property of spatial variance points out the importance of an exploratory approach to identify the best model for a given dataset (Richter and Kroschewski 2012; Richter *et al.* 2015; Sripathi *et al.* 2017). In our results, the Gaussian kernel, which is quite different from the Power kernel, best explained the underlying spatial variation in most scenarios. Identification of Gaussian kernels indicated that error deviations in neighboring plots were highly correlated due to some influencing spatial factors.

In AR, the spatial trend for the entire field is calculated as the direct product of AR from Range and Column directions (Gilmour *et al.* 1997), making this an anisotropic function. With the use of distance-based Power and other RF kernels, this status of the function can be alleviated, and it can be applied in both isotropic and anisotropic scenarios (Zimmerman and Harville 1991). Results from our real data studies indicated that nearly one-fifth of significant spatial correlation scenarios had an isotropic property (Table 2). We were able to identify and estimate the isotropy by assuming that all the plots, including those perpendicular and diagonal, are affected by the spatial trend in the field. Distance matrices were calculated based on this assumption, on which all the RF kernels were used. Use of distance-based RF kernels also remove the ambiguity on dealing the border plots (Zimmerman and Harville 1991) and presence of missing plots. Considering plot dimension in the analysis helps to correctly identify the center-to-center distance between plots. Low distance resulted in high spatial correlation between Ranges, and 92% of the directional spatial trend scenarios were in the Range direction (Table 2).

Visualization can help in identifying the pattern of gradient in the field. Knowing the pattern of spatial variation, if present, can help in mitigating the potential causal factors by modifying the management practices. For example, a continuous decrease or increase in a direction could be an indication of change in soil physical or chemical properties such as slope, texture, and structure. In other scenarios, an uneven gradient was observed. This unevenness could be because of high variation in soil due to factors such as having ridges and furrows, and fertilization variation. Visualizing and identifying the direction and magnitude of variation can help in determining the quantity and direction of irrigation, fertilization, etc. Visualization can also help in determining the size and shape of blocks or zones for precision agriculture practice (Córdoba *et al.* 2016; Yao *et al.* 2016), or even avoiding certain areas in the field for better experimental design.

Using a linear model, we were able to identify spatial trends and adjust for them to improve predictions in cassava field trials. Studies using such a modeling approach may help to understand these variations better, leading to useful changes in field operations and experimental design. Also, conducting a comprehensive field study on soil and topographic features can help to determine the causes, and, therefore, adopt specific management practices. Additionally, uniformity trials can be conducted in targeted fields to understand the spatial trends in the field (Richter and Kroschewski 2012). The linear model we proposed can also be considered as a first step analysis in estimation of genotype by environment interaction (G × E) pattern in multi-location trials (Malosetti *et al.* 2016).

### Conclusion

Through real data and simulation studies, we showed that the predictability of GS models can be improved by accounting for spatial dependency in the field. This, in turn, delivers better estimates of genotypic effect facilitating next cycle of breeding or commercialization. Use of an exploratory approach helped us to understand the best GS-spatial model in a scenario with respect to its type, direction, and gradient. Understanding these properties of spatial variation can lead to using more efficient experimental design methods, or zoning the field for precision farm practices.

## Supplementary Material

Supplemental material is available online at www.g3journal.org/lookup/suppl/doi:10.1534/g3.117.300323/-/DC1.
