# Supplementary material for: Improving Genomic Prediction in Cassava Field Experiments Using Spatial Analysis
Source: G3 (Bethesda). 2017 Nov 7;8(1):53–62. doi: 10.1534/g3.117.300323 (PMC5765366; doi:10.1534/g3.117.300323)
Supplement: Supplementary file 1 [file 53FileS1.pdf]

Table S.1: Summary of final model selected after step-wise regression of the full model using data simulated with low genotypic ratio (0.3) with all components as main effects and two-way interaction effect from simulations based on Power structure. Response variable is correlation. Model indicates different models used for analysis (Power, Spherical, and Gaussian). Fraction of spatial to total error variance is indicated by fraSp, standardization parameter by phi, and replicated versus non-replicated data by rep. The response variable as well as other variables are the same for all the tables (Tables S.1 to S.6).

```
Analysis of Variance Table

Response: COR
              Df Sum Sq Mean Sq F value    Pr(>F)    
Model          3 0.6164  0.2055  189.88 < 2.2e-16 ***
fraSp          1 0.3184  0.3184  294.22 < 2.2e-16 ***
phi            1 0.3173  0.3173  293.18 < 2.2e-16 ***
rep            1 6.2833  6.2833 5806.28 < 2.2e-16 ***
Model:fraSp    3 0.4240  0.1413  130.61 < 2.2e-16 ***
Model:phi      3 0.4737  0.1579  145.93 < 2.2e-16 ***
fraSp:phi      1 0.1340  0.1340  123.84 < 2.2e-16 ***
fraSp:rep      1 0.1380  0.1380  127.54 < 2.2e-16 ***
Residuals   2625 2.8407  0.0011                      
---
Signif. codes:  0 ***,0.001 **,0.01 *,0.05 .
```

Table S.2: Summary of final model selected after step-wise regression of the full model using dataset indicating medium genotypic ratio (0.6) with all components as main effects and two-way interaction effect from simulations based on Power structure.

Analysis of Variance Table

Response: COR

	Df	Sum Sq	Mean Sq	F value	Pr(>F)	
Model	3	0.2715	0.0905	207.5975	< 2.2e-16	***
fraSp	1	0.0095	0.0095	21.8216	3.143e-06	***
phi	1	0.0088	0.0088	20.2243	7.187e-06	***
rep	1	5.0514	5.0514	11587.9660	< 2.2e-16	***
Model:fraSp	3	0.2367	0.0789	180.9968	< 2.2e-16	***
Model:phi	3	0.1521	0.0507	116.2847	< 2.2e-16	***
fraSp:phi	1	0.0371	0.0371	85.1555	< 2.2e-16	***
Model:rep	3	0.0029	0.0010	2.2070	0.08527	.
phi:rep	1	0.0022	0.0022	4.9323	0.02644	*
Residuals	2622	1.1430	0.0004			

Signif. codes: 0 ***,0.001 **,0.01 *,0.05 .

Table S.3: Summary of final model selected after step-wise regression of the full model with all components as main effects and two-way interaction effect from simulations based on Power structure and high genotypic ratio (0.9).

Analysis of Variance Table

Response: COR

	Df	Sum Sq	Mean Sq	F value	Pr(>F)	
Model	3	0.00361	0.00120	13.9379	5.194e-09	***
fraSp	1	0.00012	0.00012	1.4186	0.2337	
phi	1	0.00427	0.00427	49.4358	2.644e-12	***
rep	1	0.55249	0.55249	6391.5393	< 2.2e-16	***
Model:fraSp	3	0.00243	0.00081	9.3823	3.644e-06	***
Model:phi	3	0.00365	0.00122	14.0762	4.256e-09	***
fraSp:phi	1	0.00180	0.00180	20.7878	5.382e-06	***
fraSp:rep	1	0.00012	0.00012	1.4143	0.2345	
phi:rep	1	0.00439	0.00439	50.8138	1.328e-12	***
Residuals	2464	0.21299	0.00009			

Signif. codes: 0 ***,0.001 **,0.01 *,0.05 .

Table S.4: Summary of final model selected after step-wise regression of the full model with all components as main effects and two-way interaction effect from simulations based on Gaussian structure and low genotypic ratio (0.3).

```
Analysis of Variance Table

Response: COR
              Df  Sum Sq Mean Sq  F value    Pr(>F)    
Model          3 15.3314  5.1105 1650.817 < 2.2e-16 ***
fraSp          1 17.9714 17.9714 5805.268 < 2.2e-16 ***
phi            1  5.4340  5.4340 1755.328 < 2.2e-16 ***
rep            1 11.8487 11.8487 3827.455 < 2.2e-16 ***
Model:fraSp    3  5.1549  1.7183  555.062 < 2.2e-16 ***
Model:phi      3  1.2031  0.4010  129.544 < 2.2e-16 ***
fraSp:phi      1  1.7120  1.7120  553.015 < 2.2e-16 ***
Model:rep      3  0.1880  0.0627   20.247 5.015e-13 ***
fraSp:rep      1  0.1521  0.1521   49.146 2.747e-12 ***
Residuals   4302 13.3177  0.0031                       
---
Signif. codes:  0 ***,0.001 **,0.01 *,0.05 .
```

Table S.5: Summary of final model selected after step-wise regression of the full model with all components as main effects and two-way interaction effect from simulations based on Gaussian structure and medium genotypic ratio (0.6).

Analysis of Variance Table

Response: COR

	Df	Sum Sq	Mean Sq	F value	Pr(>F)	
Model	3	2.9477	0.9826	1309.442	< 2.2e-16	***
fraSp	1	5.3190	5.3190	7088.550	< 2.2e-16	***
phi	1	1.1134	1.1134	1483.826	< 2.2e-16	***
rep	1	5.2493	5.2493	6995.717	< 2.2e-16	***
Model:fraSp	3	0.5730	0.1910	254.553	< 2.2e-16	***
Model:phi	3	0.2376	0.0792	105.530	< 2.2e-16	***
fraSp:phi	1	0.2475	0.2475	329.841	< 2.2e-16	***
Model:rep	3	0.0775	0.0258	34.433	< 2.2e-16	***
fraSp:rep	1	0.0457	0.0457	60.844	7.911e-15	***
phi:rep	1	0.0080	0.0080	10.711	0.001074	**
Residuals	3821	2.8671	0.0008			

Signif. codes: 0 ***,0.001 **,0.01 *,0.05 .

Table S.6: Summary of final model selected after step-wise regression of the full model with all components as main effects and two-way interaction effect from simulations based on Gaussian structure and high genotypic ratio (0.9).

```
Analysis of Variance Table

Response: COR
              Df  Sum Sq Mean Sq   F value  Pr(>F)    
Model          3 0.18479 0.06160  500.7850 < 2e-16 ***
fraSp          1 0.14661 0.14661 1191.9521 < 2e-16 ***
phi            1 0.07193 0.07193  584.8153 < 2e-16 ***
rep            1 1.01630 1.01630 8262.5284 < 2e-16 ***
Model:fraSp    3 0.04054 0.01351  109.8724 < 2e-16 ***
Model:phi      3 0.01920 0.00640   52.0426 < 2e-16 ***
fraSp:phi      1 0.01511 0.01511  122.8137 < 2e-16 ***
Model:rep      3 0.01243 0.00414   33.6895 < 2e-16 ***
phi:rep        1 0.00038 0.00038    3.0776 0.07945 .  
Residuals   4302 0.52915 0.00012                      
---
Signif. codes:  0 ***,0.001 **,0.01 *,0.05 .
```

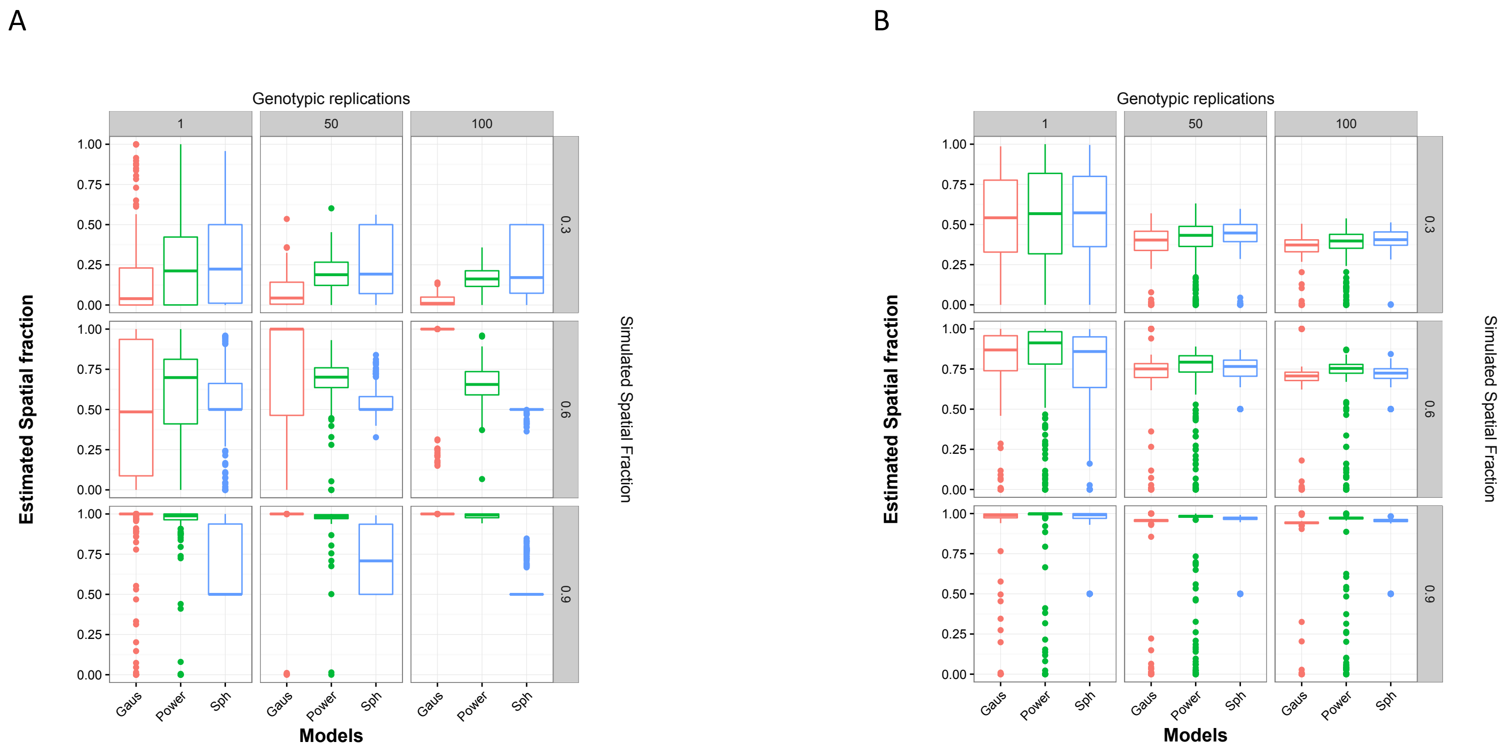

Figure S.1: Estimated spatial fraction from data simulated with A) Power and B) Gaussian correlation functions. Simulated fraction of spatial to total error variance is given in horizontal panels and number of replications for test genotypes (1 indicates that no test genotypes were replicated, 50 and 100 indicates 50% and 100% of the test genotypes were replicated) is given in vertical panels.

A.1 – Ibadan_2013_C1 - DM

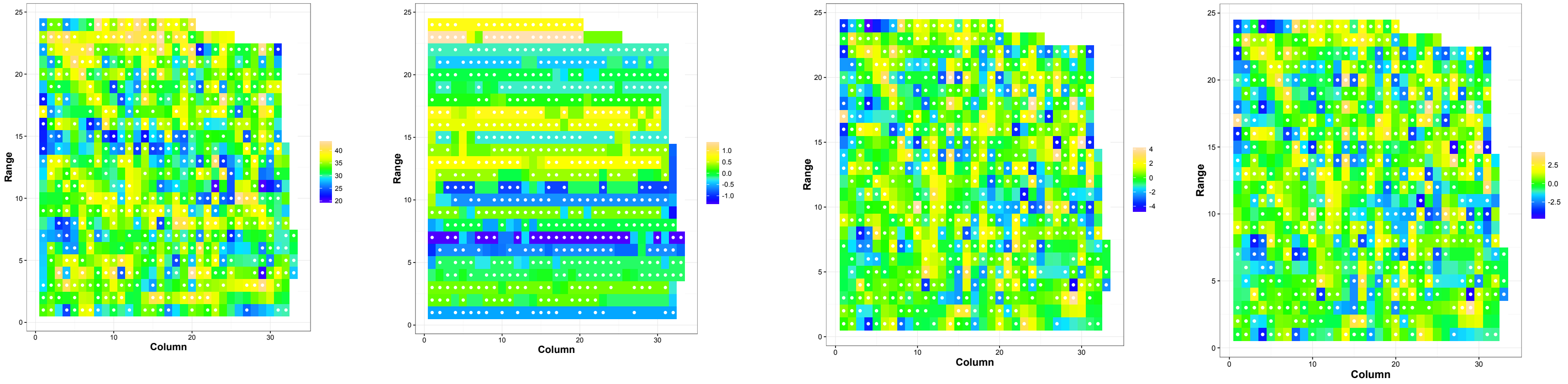

A.2 – Mokwa_2014_C2 - DM

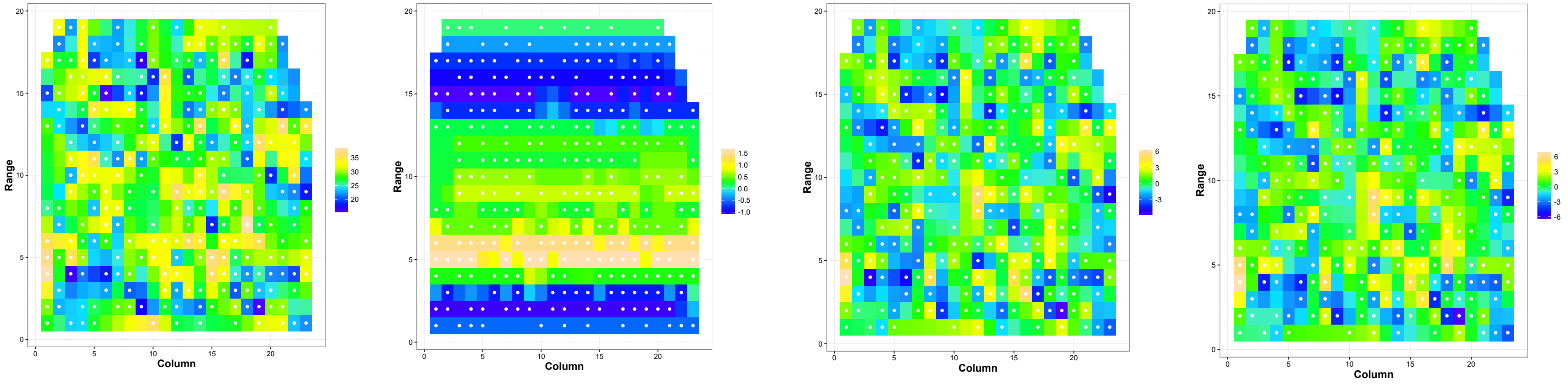

A.3 – Ikenne_2013_C1 - DM

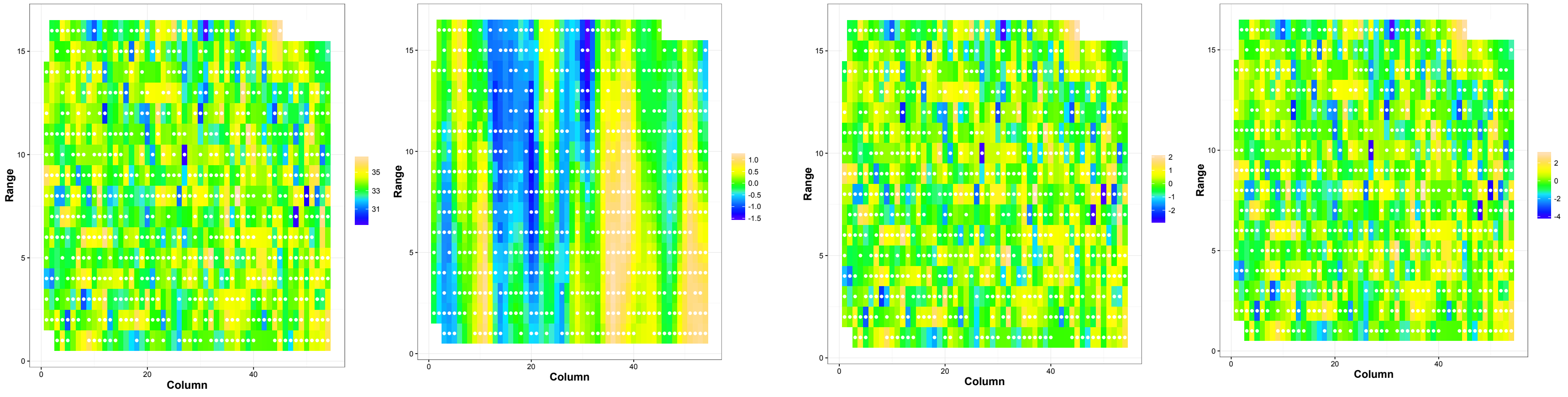

A.4 – Mokwa_2013_C1 - DM

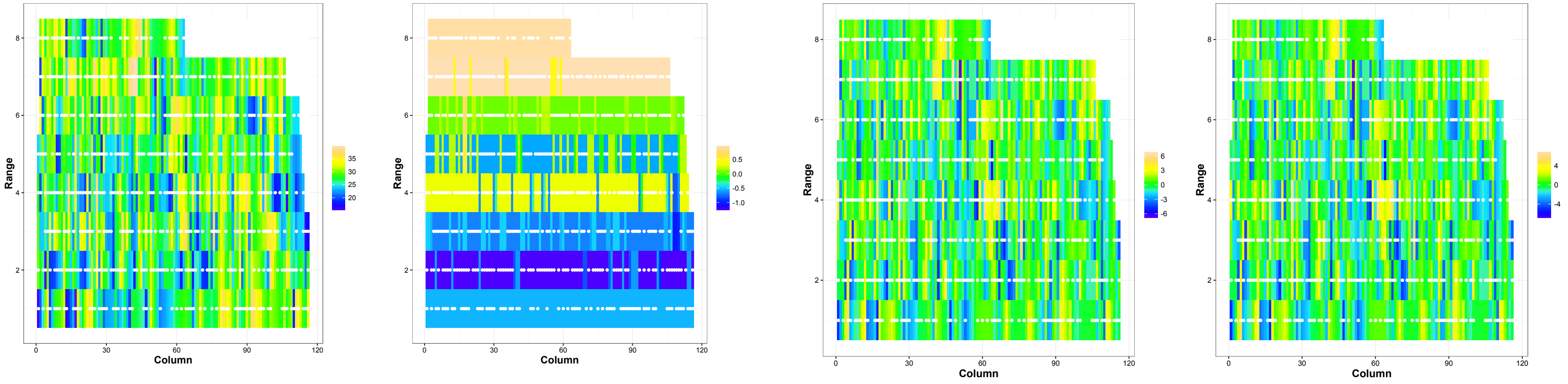

B.1 – Mokwa_2014_C2 - HI

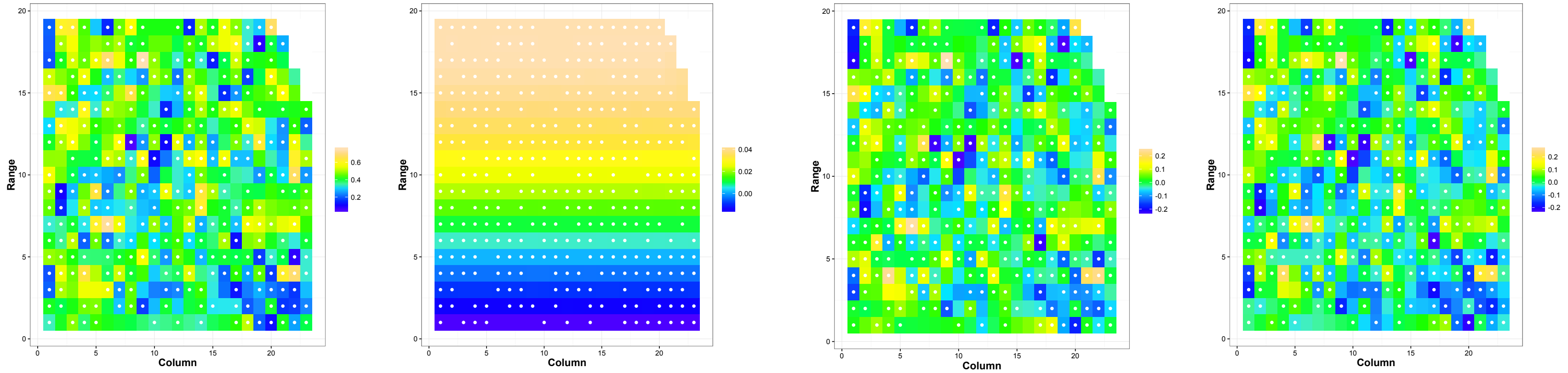

B.2 – Ibadan_2014_C1 - HI

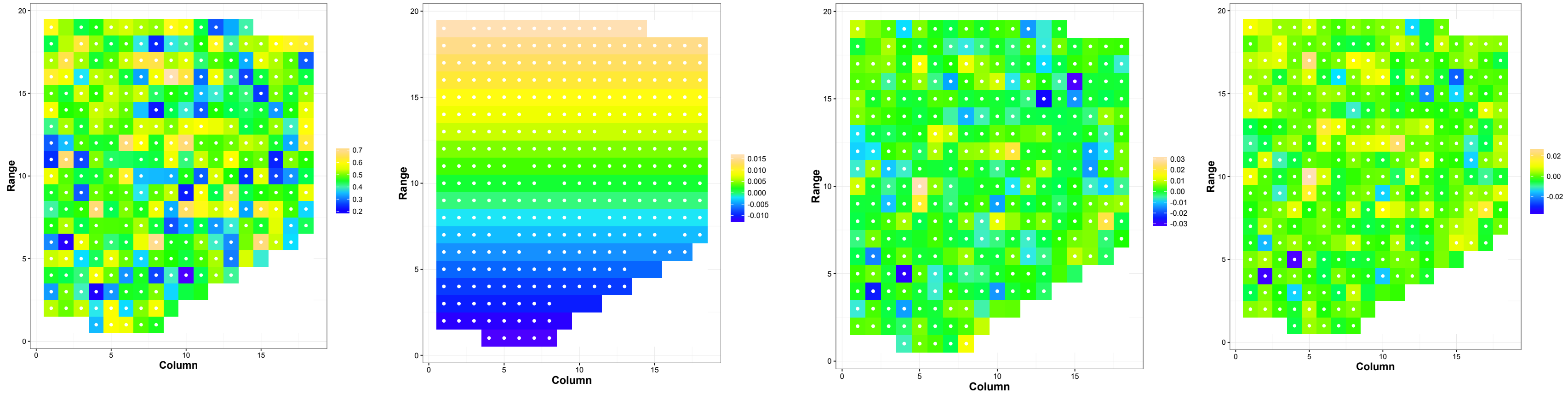

B.3 – Ikenne_2013_C1 - HI

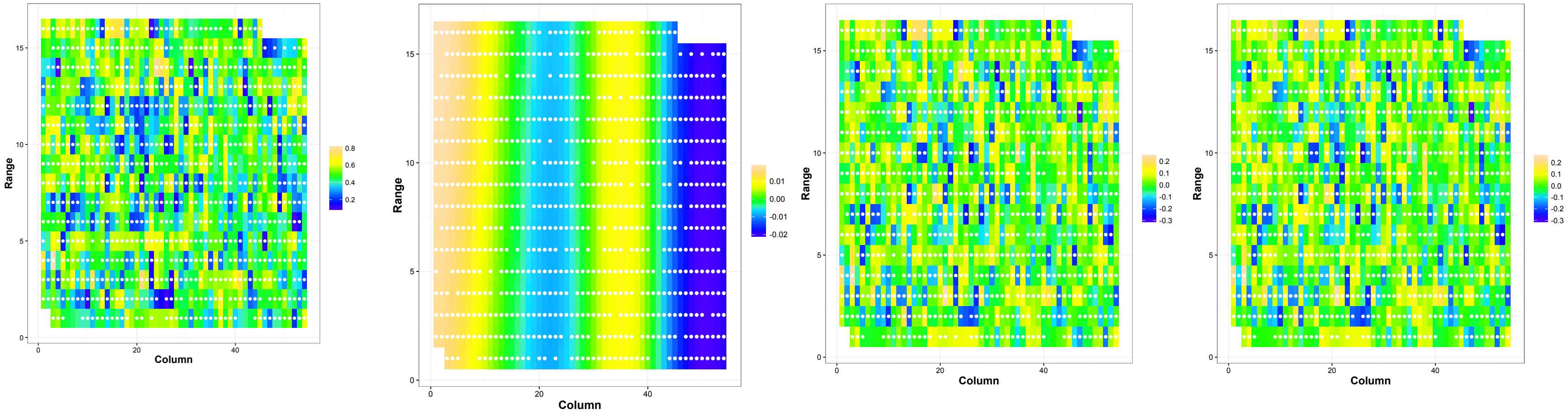

B.4 – Mokwa_2013_C1 - HI

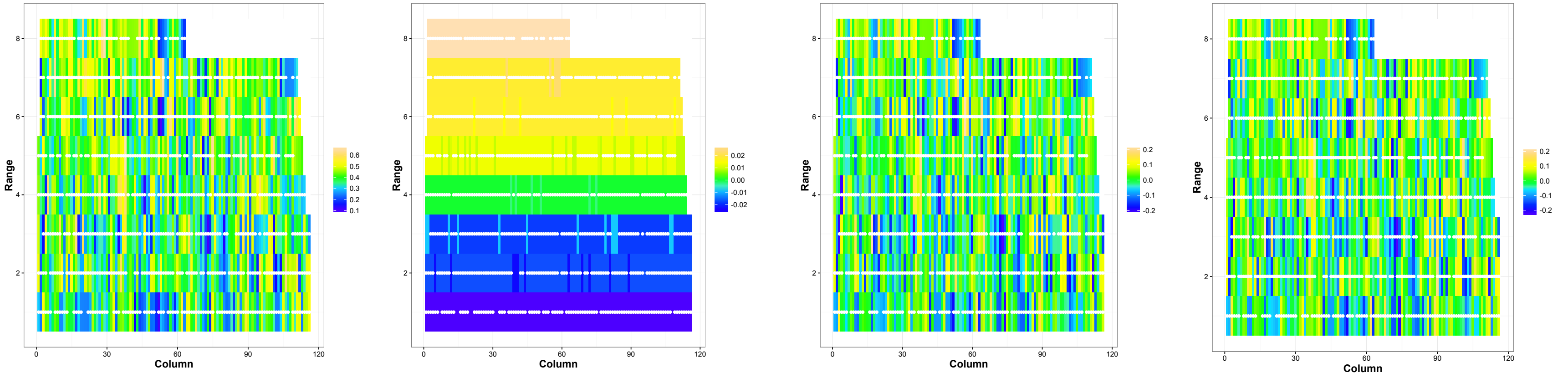

C.1 – Ibadan_2014_C1 - FYLD

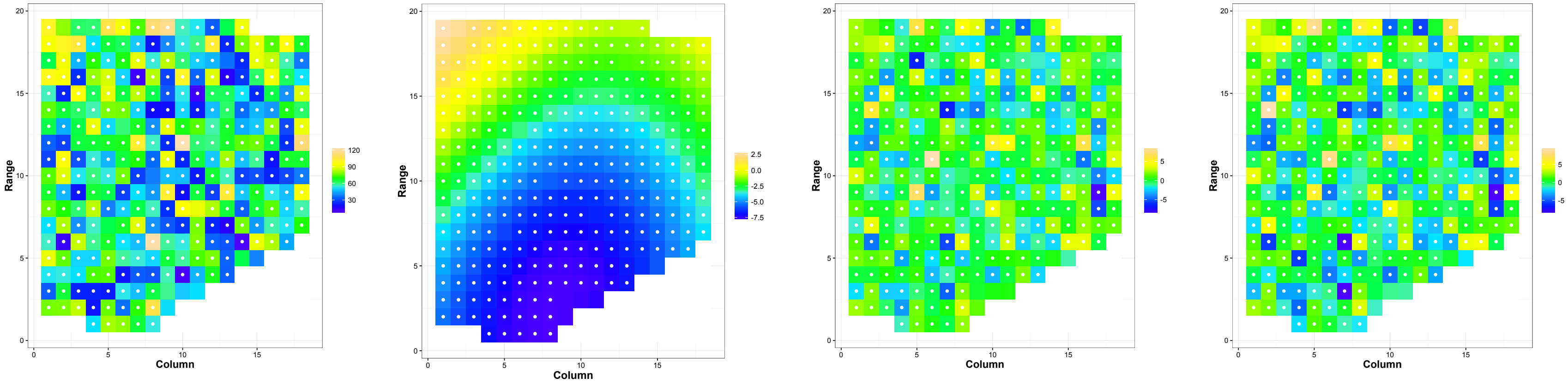

C.2 – Mokwa_2013_C1 - FYLD

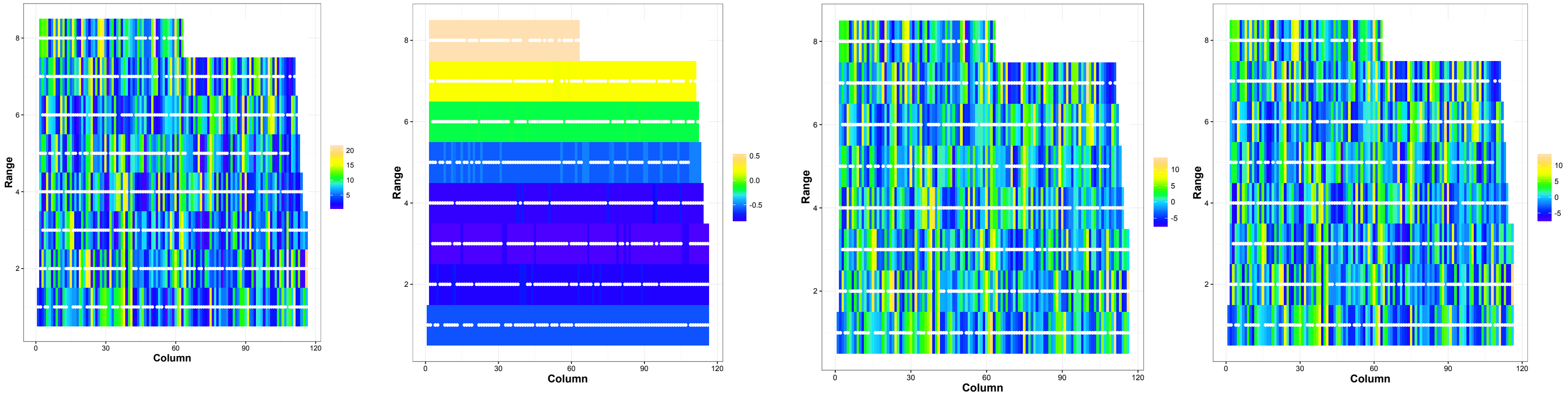

D.1 – Ibadan_2013_C1 - SHTWT

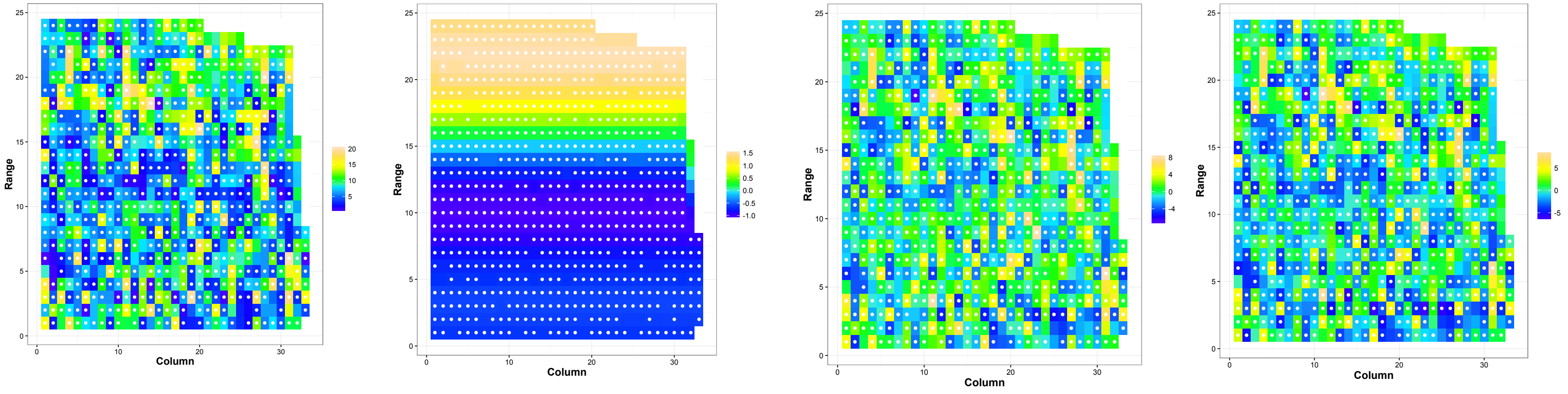

D.2 – Mokwa_2014_C2 - SHTWT

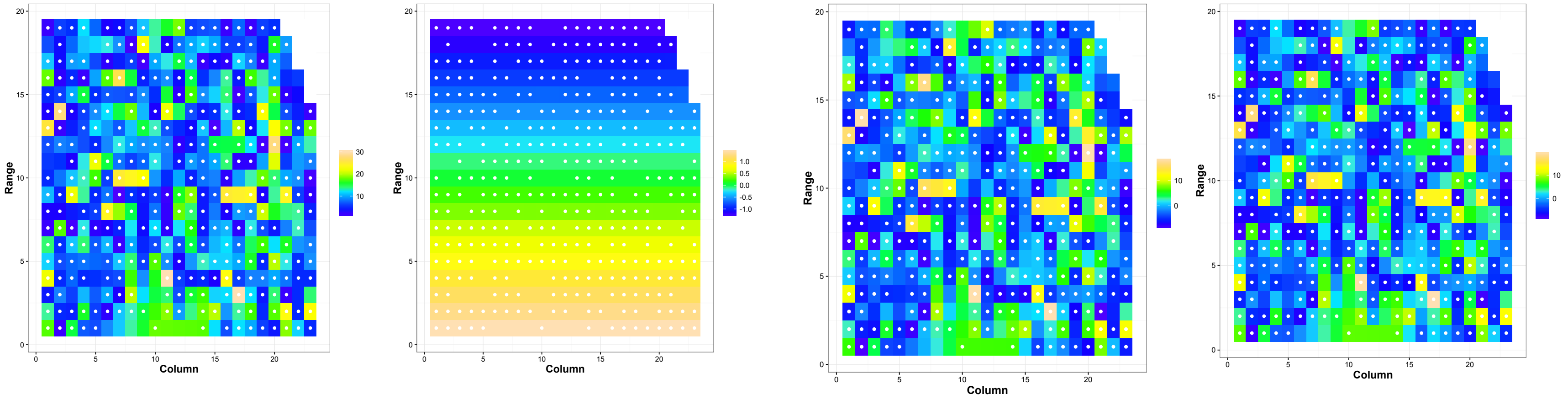

D.3 – Ibadan_2014_PYT - SHTWT

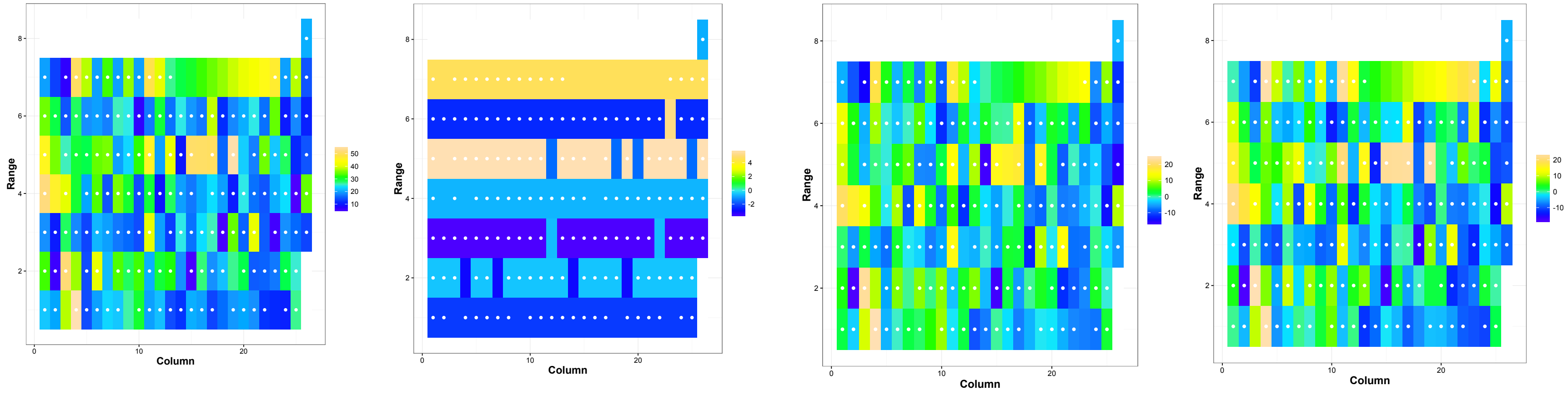

D.4 – Ikenne_2013_C1 - SHTWT

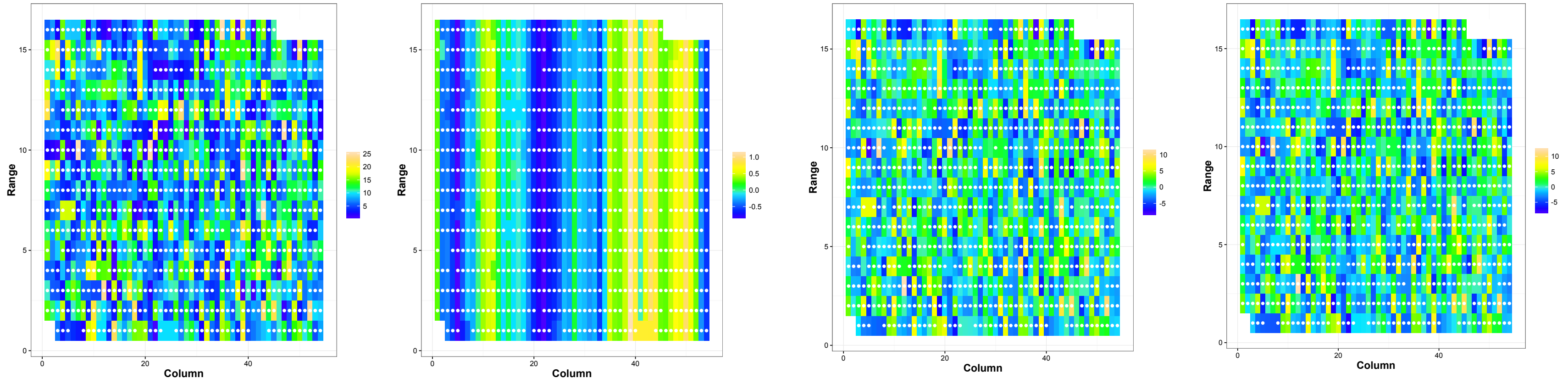

Figure S.2: Original observation (column 1), spatial BLUP (column 2), residual from Model 1 (column 3), and residual from Base (column 4) for A) root dry matter (DM), B) harvest index (HI), C) fresh root weight (FYLD), and D) fresh shoot weight (SHTWT). Depicted below are the trials/traits which exhibited significant spatial correlation: A.1) Ibadan_2013_C1, A.2) Mokwa_2014_C2, A.3) Ikenne_2013_C1, A.4) Mokwa_2013_C1, B.1) Mokwa_2014_C2, B.2) Ibadan_2014_C1, B.3) Ikenne_2013_C1, B.4) Mokwa_2013_C1,

C.1) Ibadan_2014_C1, C.2) Mokwa_2013_C1, D.1) Ibadan_2013_C1, D.2) Mokwa_2014_C2, D.3) Ibadan_2014_PYT, and D.4) Ikenne_2013_C1. Plots are rectangular and white dots inside each plot indicate the plots whose observations were used. For representation, plot lengths and widths are not equally scaled. Missing values are interpolated linearly. Though spatial patterns are clear, no striking difference in the pattern of residuals can be noticed because spatial effects were small relative to the total error.
